# How to prevent, minimize, or extinguish nocebo effects in pain: a narrative review on mechanisms, predictors, and interventions

**DOI:** 10.1097/PR9.0000000000000699

**Published:** 2019-06-07

**Authors:** Meriem Manaï, Henriët van Middendorp, Dieuwke S. Veldhuijzen, Tom W.J. Huizinga, Andrea W.M. Evers

**Affiliations:** a Health, Medical and Neuropsychology Unit, Leiden University, Leiden, the Netherlands; b Leiden Institute for Brain and Cognition, Leiden University, Leiden, the Netherlands; Departments of c Rheumatology and; d Psychiatry, Leiden University Medical Center, Leiden, the Netherlands

**Keywords:** Nocebo, Conditioning, Verbal suggestion, Pain, Placebo

## Abstract

Nocebo effects, such as side effects due to negative expectations regarding the pain treatment, are a concern for health care providers and come with significant costs. This narrative review focuses on underlying mechanisms and possible factors that contribute to the susceptibility to the nocebo effect on pain and related outcomes and suggests strategies that can prevent, minimize, or extinguish nocebo effects in clinical settings. Nocebo effects are the result of psychological (eg, conditioning, verbal suggestions, and observational learning) and neurobiological (eg, cholecystokinin and dopamine regulation) mechanisms. Evidence from clinical and experimental studies lead to various recommendations and strategies to alter the nocebo effect in order to optimize pain treatments, such as providing patients with enhanced information, optimizing patient–physician communication and relationships, and offering psychoeducation on coping skills in order to manage patient expectations. The current literature from both clinical and experimental studies provides a better understanding of the nocebo effect and possible factors that modulate its strength on treatment outcomes. This allows for the development of evidence-based strategies aimed at the prevention, minimization, and treatment of the nocebo effect in pain conditions and possible other somatic disorders.

## Abstract 2

Possible factors that contribute to nocebo effects on pain are explored. Strategies that can prevent, minimize, or extinguish nocebo effects in clinical settings are suggested.

## 1. Introduction

In clinical trials and practice of pain treatments, there is a large interindividual variability in treatment outcome between patients.^5^ One important factor influencing the interindividual variability is the role of expectations, which can optimize or add to analgesic treatment outcomes in case of positive expectations (so-called placebo effects) or worsen pain or cause for inadequate pain relief in case of negative expectations (so-called nocebo).^26,29,97,123^ A growing body of literature is focusing on how the positive effects that can be reached by using placebo effects can be optimized for use in clinical practice.^2,39,58,102,103^ Much less attention has been paid to the question on how to prevent, minimize, or extinguish negative expectations by means of the nocebo effect in clinical practice. However, since negative or nocebo expectations (eg, “The pain will worsen,” “I always react hypersensitive to medication”) have shown even stronger effects than positive or placebo expectations, possibly due to the evolutionary determined bias for negative threatening information, looking into ways to diminish the nocebo effect is highly relevant.^8^

The nocebo effect is an adverse effect to a treatment that cannot be ascribed to a specific treatment mechanism.^105^ Instead, this effect is caused by the expectations that a person has about the effects of the treatment.^5,11,63^ Examples of the nocebo effect include the reporting of side effects in the placebo condition of randomized controlled trials,^34,98,109^ the experience of a pain increase in response to the doctor saying that a painful procedure will take place,^14,25,30,127^ or becoming nauseous when entering the hospital where one receives chemotherapy.^5,22,115^

Because nocebo expectations lead to adverse effects, such as negative treatment outcomes, they can contribute to significant costs of (chronic) clinical conditions in the form of decreased quality of life, treatment nonadherence, financial costs for health services, and societal costs.^126^ For this reason, it is highly relevant to prevent, minimize, or extinguish nocebo effects in (chronic) pain conditions. Therefore, the current article will provide a narrative review on nocebo applications in pain and related conditions in both experimental and clinical settings, to identify factors that may cause increased susceptibility to the nocebo effect and to formulate concrete and to specific situations tailored recommendations on how to prevent, minimize, or extinguish the nocebo effect in clinical practice.

## 2. Learning mechanisms of nocebo effects

**Figure 1.**
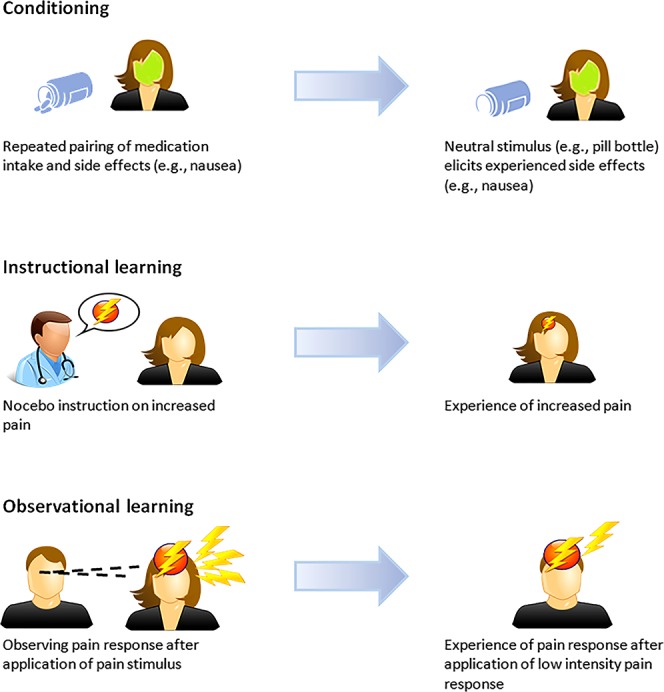
Learning mechanisms of the nocebo effect.

Expectations play a pivotal role in placebo and nocebo effects for pain and related conditions.^49,64,82^ Learning theories explain that expectations, in particular response expectancies, can be acquired in various ways, including conditioning, instructional learning through verbal suggestions regarding treatment effects, and observational learning,^6,7,10,78,82^ see also Figure 1. These mechanisms do not only exert influence on behavior, but also on nonvolitional physiological responses.^27,89^ In conditioning, new associations are formed by repeatedly pairing 2 stimuli, whereby specific conscious or automatic expectations (eg, nausea due to chemotherapy^3^) or latent responses (eg, conditioned immune responses^39^) are induced. Research in both experimental and clinical settings has shown that these repeated pairings between a neutral stimulus and a physiological outcome can result in conditioned nocebo effects.^3,5–7,15,34,98,99,109^ Because of the conditioning process whereby associations are formed between a treatment and its outcome and possible side effects, patients who have had previous negative experience with a treatment are more prone to experience nocebo effects when starting a new treatment. Previous experience with side effects may also result in the attribution of common maladies to medication side effects.^5,28,31,32^ For instance, previous experience with chemotherapy will cause for nocebo nausea in a substantial number of patients.^1,3,83,129^ In addition, infants receiving repeated heal lances in the first 24 to 36 hours of life to monitor blood glucose levels showed more intense pain responses than infants receiving only one painful procedure. Furthermore, the skin cleaning procedure that infants underwent for each heal lance resulted in anticipatory pain behaviors, indicating that the cleaning procedure became a conditioned stimulus.^114^ These studies suggest that nocebo effects can be induced by environmental stimuli associated with the treatment, such as the sight of a familiar health care worker, the sounds and smells of the treatment context, the sight of the color and shape of the medication itself, or a cleaning procedure.^5,114^ Conditioning subsequently reinforces and generalizes the side effects that patients experience by a process of sensitization and can be strengthened even further with the number of conditioning sessions.^31^

In addition to learned associations, instructional learning, such as verbal information about negative treatment outcomes, can influence somatosensory perception and can result in symptom aggravation.^14,32^ This is evident in patient–physician communication whereby the information on the risks of a possible treatment can induce negative expectations and treatment anxiety, resulting in nocebo effects.^5,14,25,30,129^ For example, in a trial studying the effects of pharmacological treatments of unstable angina, patients who were informed about possible gastrointestinal side effects of the treatments withdrew 6 times more often due to minor gastrointestinal complaints than patients who did not receive this information.^84^ Nocebo effects have also been induced in pain and other conditions by giving negative verbal suggestions about the expected outcome after a clinical treatment or experimental manipulation.^6,14,15,25,30,118^

Robust empirical evidence shows that nocebo effects can be caused by conditioning, verbal suggestion, or a combination of the two.^14,28,32,37,90,105,126^ Although placebo research indicates that learned associations due to previous experience with a treatment (conditioning) can cause stronger placebo responses than verbal suggestions and that the combination of a conditioning procedure with verbal suggestions results in additive placebo effects,^95^ this is not necessarily the case for nocebo effects.^24,32^ For instance, one study in line with the placebo literature showed that nocebo itch was only induced in a group that underwent a conditioning procedure in combination with verbal suggestions about the intensity of the itch stimuli compared with groups that underwent these procedures separately or a control group.^6^ By contrast, another study found no differences between either conditioned pain or pain induced by verbal suggestions, suggesting that conditioning is less dominant in evoking nocebo than placebo responses.^32^ However, in the latter case, this could also be due to the type of stimulus applied in this study, namely electric shock. Here, the stimulus can easily be interpreted as dangerous, and therefore, it does not necessarily need previous experience to perceive the stimulus as a potential painful threat.^32^

Finally, strong nocebo effects can also be evoked through a third learning mechanism, observational learning, which can even be more robust than verbal suggestion about possible negative effects.^10,48,88,123^ It has, for example, been shown that watching a person with intensified pain after the application of an ointment led to larger increases in pain ratings in response to a subsequent pain stimulus than verbal suggestions that the ointment increased pain intensity.^123^ The finding that observational learning can elicit nocebo effects suggests that first-hand experience through conditioning or verbal suggestion are not the only key factors in the nocebo phenomenon, but that social learning and interaction play essential roles as well.^10,48,88^

These studies indicate that several psychological learning mechanisms are at play in the nocebo effect and may provide different avenues for the development of possible treatments that target nocebo effects. However, the combination and interaction of the various mechanisms need further investigation to gain insight in how to be optimally used.

## 3. Neurobiological mechanisms of the nocebo effect

Neuroimaging studies have provided evidence that the anticipation of pain, due to negative pain expectations, activates several brain regions involved in processing nociceptive stimuli. These include the prefrontal cortex, the thalamus, the secondary somatosensory cortex, the anterior cingulate cortex, the parietal operculum, and the insular cortex.^18,37,54,61,67,74,92–94,101,104,105^ Furthermore, the expected intensity of a noxious stimulus increases activation in the thalamus, the prefrontal cortex, the anterior cingulate cortex, the head of the caudate, the cerebellum, and the contralateral nucleus cuneiformis.^61,67^

Neurochemical pain research indicates that anticipatory anxiety, also associated with negative expectations, activates at least 2 independent pathways, namely the hypothalamus–pituitary–adrenal (HPA) axis, which controls reactions to stress,^68^ and the cholecystokinin (CCK-ergic) system, which is involved in the regulation of nociception, anxiety, and memory.^37^ In this nocebo pain model, the HPA axis is activated by nocebo suggestions and results in anticipatory anxiety. This has been shown in various studies that saw an increase in both cortisol and adrenocorticotropic hormone after verbally induced nocebo hyperalgesia.^13,56^ After the administration of diazepam, a benzodiazepine anxiolytic drug, both HPA activation and pain perception were reduced, confirming the role of (anticipatory) anxiety in nocebo hyperalgesia.^13^ In turn, this anticipatory anxiety activates the CCKergic system, which activates descending pronociceptive pathways from the periaqueductal gray and mediates anxiety-induced hyperalgesia.^13,65,75^ Indeed, infusion of the CCK-blocking drug proglumide reverses the nocebo effect.^12,13^ In addition, increasing research focuses on the interaction between CCK and opioids and dopamine (DA) because it has been found that CCK acts as a neuromodulator of pain, whereas opioids and DA have pain-relieving properties.^9,107^ Similarly, CCK can be activated by verbal suggestions of pain increase (nocebo), whereas endogenous µ-opioid neurotransmitters and DA can be activated by suggestions of pain decrease (placebo).^107^ Finally, because CCK antagonizes opioid effects,^9^ the CCK-induced nocebo effects deactivate opioid and DA release.^107^ These findings open up new avenues of pharmacotherapeutic strategies for pain treatment when anxiety is involved. Namely, CCK-blocking pharmacotherapeutics could be applied to limit anticipatory anxiety, with or without combining these with positive verbal suggestions, to activate neurochemical placebo responses in the form of µ-opioid and DA.

In summary, several neurobiological and neurochemical trajectories are suggested to work independently and interact to produce nocebo effects. However, further research is needed to gain a better understanding of both their separate and their overlapping roles for possible additive and interactive effects.

## 4. Susceptibility to the nocebo effect

The nocebo effect is formed by multifaceted factors that relate to the individual and interact with the environmental context of clinical and laboratory settings.^5^ Examples of individual characteristics that can influence the magnitude and the prevalence of the nocebo effect include genotype variations, age and sex, personality characteristics, and psychological distress.

### 4.1. Genetics

Research indicates that genetic variations can influence the nocebo effect.^21^ An example is the rs4680 single-nucleotide polymorphism, which is a well-studied gene for catecholamines-O-methyltransferase (COMT) that metabolizes DA and other catecholamines.^69^ This gene codes a valine (val) amino acid to a methionine (met) at codon 158 (val158met). Homozygotes for the less-active rs4680 met allele (met/met) metabolize DA at a lower rate and showed a larger placebo response than homozygotes for the highly active val allele (val/val). The latter population not only shows smaller placebo responses, but also reports more nocebo symptoms.^51,69,128^ Coincidentally, research found that, compared with the other genotypes (heterozygotes and met/met homozygous carriers), homozygote carriers of the val158 have a higher disposition to detect somatic and visceral sensations and experience these sensations as strong, unpleasant, and harmful. Furthermore, people with val/val genotypes believe more strongly that medication will harm them, are more sensitive to their effects, and are more concerned about possible side effects of medication.^128^ Because evidence suggests that genotype variations involved in DA modulation may influence the nocebo effect, other polymorphisms may exert their influence on this reward pathway as well. For instance, the rs6323 single-nucleotide polymorphism for monoamine oxidase A (MOA-A) not only metabolizes monoamines, but also serotonin.^80^ Previous research has shown that increased serotonin levels stimulate the placebo response but counteract the nocebo effect in patients with Parkinson disease.^15^ Although these studies provide knowledge of the possible moderators of the nocebo effect, it should be considered that our knowledge in this area is still limited.

### 4.2. Age and sex

Studies that focused on the role of age have not yet shown a significant role of age for the susceptibility to the nocebo effect.^34,45,47,73,131^ In addition, most research on nocebo effects shows that sex does not significantly contribute to the nocebo effect.^126^ However, some studies did find that women are more susceptible for nocebo effects than men.^17,72,91,112,113^ For instance, a study^66^ investigating the effect of sex on conditioning or verbal suggestions in healthy adults found that women showed a larger nocebo effect after induced nausea than men. Furthermore, women were more affected by conditioning than by verbal suggestions, whereas men responded stronger to verbal suggestions. This could indicate that finding sex differences in the susceptibility to the nocebo effect is dependent on the way the nocebo effect is induced. These potential sex differences might also be mediated by cognitive variables. For instance, research suggests that women may have more stable negative outcome expectancies because correlations indicate that women tend to worry more than men^71,77,100,110^ and are possibly more oriented towards problems.^100^ Future research is needed to gain knowledge on whether the interaction between sex and cognitions may have an influence on nocebo effects in acute situations such as in an experimental setting or in clinical practice during physician consultations. In addition, although the influence of age and sex on nocebo effects in various clinical populations with diverse symptomologies has been studied,^126^ few studies are available that studied their influence on nocebo pain specifically. Therefore, further exploration of these influences on nocebo effects for pain would add significantly to the literature.

### 4.3. Types of pain

Nocebo pain has been well studied in instances of acute nociceptive pain, for example, in experimental settings whereby a noxious stimulus is applied or in patient populations suffering from postoperative pain.^120^ Furthermore, randomized controlled trials investigating active drugs compared with a placebo arm have expanded the knowledge on nocebo responding to placebo treatment in, for example, neuropathic pain.^43^ However, because studies that involve nocebo effects in randomized controlled clinical trials usually compare an active drug with a placebo arm, but not to a no-treatment control arm, the natural history of pain is not controlled for. This makes it difficult to infer whether nocebo effects are actually due to nocebo components or other factors, such as regression to the mean. Furthermore, nocebo pain may differ depending on the setting, being either experimental or clinical. However, no strong conclusions can be drawn regarding this potential difference because of the heterogeneity in populations and selection of outcome measures (eg, pain, itch, or nausea). Few studies^120^ have attempted to elucidate what roles different types of pain (eg, nociceptive, neuropathic, acute, or chronic pain) play in the nocebo effect, and more research is needed to gain conclusive knowledge on the potential separate and overlapping underlying mechanisms of different types of pain in nocebo responding or the setting in which nocebo pain takes place (experimental vs clinical).

### 4.4. Type of medication

Not much research has investigated whether the type of medication received by the patient can significantly contribute to the nocebo effect. A recent meta-analysis^121^ indicated that opioid trials are correlated with higher placebo responses than nonopioid pain medication. This may be due to several factors. First, opioids are established as potent analgesics, and patients may therefore have pre-established expectations on their efficacy. Second, research^121^ indicates that opioid trials are also accompanied by a higher number of face-to-face visits, which may have an influence on instructional learning, because there is a higher chance of verbal instructions on the opioids' efficacy being repeated, thereby strengthening expectations. Although these findings indicate that the type of drug associated with nocebo effects may have an influence on nocebo responding, more research is needed.

### 4.5. Personality characteristics

Although more research is needed to clarify the role of personality on nocebo effects, certain personality traits have been linked to the susceptibility to the nocebo effect. For instance, although optimists seem to be more responsive to the placebo effect,^33,46^ pessimists may be more vulnerable for nocebo effects.^33,45^ In addition, individuals with type A personalities, who can be described as more aggressive, competitive, and impatient,^16,36^ are more likely to report side effects than type B individuals,^36^ who may be less aggressive and more easy going.^16,36^ Lower levels of extraversion, which can be construed as being more outgoing and talkative,^42^ have also been indicated as a possible contributing factor in the nocebo effect.^6^ Anxiety, psychological suggestibility, and worrying seem to significantly correlate with nocebo effects.^6,33,106,122^ Patients with rheumatoid arthritis were found to be at greater risk of developing treatment side effects if they worried more about their medication intake, especially when starting a new treatment.^85,86^ Patients who worry more about developing negative side effects may be more attuned to adverse symptoms and may interpret any sensation, whether new or preexisting, to their medication intake, while at the same time ignoring the positive effects of a treatment.^5^ Mixed results have also been found for imaginative involvement, which can be described as the ability to experience suggestions imaginatively, where one study found that higher levels of imagination were related to stronger nocebo effects on itch,^106^ whereas another study did not find any significant correlations for imaginative involvement in nocebo itch induced by verbal suggestions and pain responses.^118^

Certain personality traits may predispose individuals to the nocebo effect and insight into their contribution to nocebo responding allowing for the tailoring of evidence-based strategies to minimize or manage the nocebo effect in clinical settings to specific subgroups of patients most vulnerable to nocebo effects. However, more empirical investigation is needed because research in this area is not yet comprehensive and conclusive.

### 4.6. Psychological distress

Patients with symptoms of depression seem to be more prone to developing nocebo side effects. It has been theorized that these patients are more focused on somatic sensations, are more inclined to expect negative outcomes, and may even feel that they deserve negative outcomes.^5^ Furthermore, patients with conditions such as depression or anxiety have a tendency to somaticize, which has been shown to result in increased reports of side effects.^5,76^ For example, patients with rheumatoid arthritis who had higher baseline reports of somatization and a tendency to exaggerate negative somatic sensations were more likely to develop nocebo effects.^116^ Negative side effects reported by highly anxious patients tend to be similar to symptoms of anxiety itself, such as tachycardia, dyspnea, and sweating.^91^ Thus, factors related to psychological distress, such as symptoms of depression and anxiety, may contribute or aggravate susceptibility to the nocebo effect by emphasizing the focus on negative somatic symptoms.

The influence on the nocebo effect of these person-related factors and the manner in which they interact with the social and environmental context suggests that these multifaceted individual factors may be deployed in the manipulation of nocebo effects.

## 5. Challenges and opportunities: recommendations to prevent, minimize, and extinguish the nocebo effect in clinical practice

Because the occurrence of the nocebo effect has been established in a wide variety of clinical populations,^5,14,25,28,65,90,105^ it is essential to develop strategies that can prevent, minimize, or extinguish its adverse effects. Several factors contribute to the strength of the nocebo effect, such as patient–physician relationship and physician communication style.^25,30^ These contributing factors can be used as a basis on which recommendations can be formulated to prevent, minimize, or extinguish the nocebo effect. For example, screening instruments could be implemented to identify personalities that may have an increased risk for nocebo responding. Based on the outcome of the screening, pharmacological treatment could be supplemented with psychological strategies that help manage physical, psychological, and social functioning. Based on the current literature,^25,28,30,40,65,125^ evidence-based ethical recommendations can be developed to optimally treat the nocebo effect in clinical practice, see also Table 1.

**Table 1:**
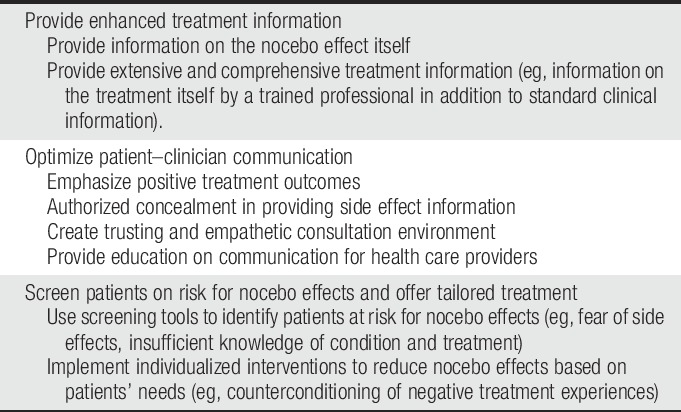
Strategies to prevent, minimize, and extinguish the nocebo effect in clinical practice.

### 5.1. Enhanced treatment information

Because verbal and nonverbal communication can induce negative treatment expectations that may result in nocebo effects,^5,52^ patient–clinician communication is of utmost importance. Even when negative associations are formed based on one encounter with negative information in the form of verbal suggestions or informational leaflets, without the process of conditioning, a once established nocebo effect can be long-lasting.^14,25,65,101^ Often in clinical practice, the limited time that clinicians have during appointments with patients leads to a shift in the priority to giving patients very concise information regarding a new treatment, with a focus on possible side effects of a treatment as opposed to emphasizing the potential positive effects of a treatment. The clinical environment can therefore unintentionally induce nocebo effects.^65,129^ Providing enhanced information about the nocebo effect itself and the prescribed treatment could prevent the induction of nocebo effects.^40,119^ Giving enhanced treatment information by a trained professional in addition to standard clinical information may lead to higher satisfaction and knowledge about the illness and its treatment.^53^ A better understanding of the prescribed treatment may exert a positive influence on patients who show concerns about medication intake and medication dependence.^55^ As these types of worries may exacerbate the nocebo effect, providing extended treatment information may be a strategy in preventing or minimizing nocebo responding.

### 5.2. Optimization of patient–clinician communication

Verbal suggestions are a powerful strategy to enhance negative expectations and can induce or strengthen nocebo effects. Therefore, the information that physicians provide their patients on the treatment and its possible side effects could induce strong nocebo effects. For this reason, treatment information can be conveyed in such a way that positive treatment effects are emphasized, while avoiding the overemphasis on treatment side effects.^30,65^ In this positive framing method, an ethical balance should be maintained to minimize clinician-induced nocebo effects through verbal suggestions on possible treatment side effects, while simultaneously respecting patient autonomy, because clinicians have an obligation to thoroughly inform their patients on important treatment information.^38^ Wells et al.^127^ therefore suggest that clinicians apply contextualized informed consent, which involves giving tailored information about medication side effects while considering various components, such as the person, the disease under treatment, and the possible side effects of the treatment. However, because physicians have an ethical duty to provide full treatment disclosure, Colloca^23^ suggests informing patients on the nocebo phenomenon and asks if patients would prefer to not be informed about possible side effects of a treatment and essentially provide authorized concealment. If the patient does prefer to receive full disclosure, a physician may ask the patient to contact her/him if any new or unusual symptoms should arise instead of listing the nonspecific side effects of a treatment. In addition, talking about the chances of not experiencing side effects is preferred over giving information on the chances of experiencing possible side effects.^126^ An example is provided by a study^87^ in which unimmunized patients with chronic respiratory or cardiac disease were either informed about the percentage of recipients of the influenza vaccine who remain free of influenza and do not experience vaccine side effects, or received information on the percentage who do acquire influenza and suffer from vaccine side effects. The group that received positively framed information reported fewer side effects and less work absenteeism.^88^

Research suggests that health care providers could receive education on how nocebo effects can influence clinical outcomes and how to convey information in such a way to prevent or minimize nocebo responding from occurring.^40^ In turn, physicians could convey information on the nocebo effect to their patients and either withhold negative treatment information (authorized concealment) or incorporate a positive framing style of communication.^23,28,65^ Other evidence-based recommendations to improve patient–clinician communication include to identify patient expectations and possible fears, and to evaluate the patient's understanding of the diagnosis and suggested treatment.^59^ This could be especially important in patient populations that are highly susceptible to the nocebo effect,^108^ such as people with negative previous treatment experience or people who worry more.^126^ Optimizing the communication style in clinical settings could be an important approach in preventing or minimizing nocebo effects.

### 5.3. Optimization of patient–clinician relationship

Not only negative information itself could lead to nocebo effects but also nonverbal communication and communication style, such as keeping a medical consult formal and not providing a trusting and empathetic environment,^5,35,52,59^ as research has shown that the manner in which a physician is perceived can influence the effectiveness of a treatment.^59,60,111^ Clinicians who convey a sense of warmth, friendliness, and reassurance obtain more effective treatment effects than clinicians who keep their consults formal without any form of reassurance. An effective patient–clinician relationship involves mutual trust, empathy, respect, genuineness, acceptance, and warmth.^35,59^ Because negative associations between the clinical context and a treatment could elicit strong nocebo effects, it is preferred to create an environment that is associated with positive expectations rather than feelings of fear and uncertainty. This is especially important for health care workers who have direct patient contact as merely seeing them could elicit nocebo effects.^5,35,59^ Therefore, physician educational strategies that focus on communication skills that promote trust, mutual understanding, adherence, social support, and self-efficacy could foster positive expectations about the suggested treatment outcome and minimize or even prevent nocebo effects.^40,111^

### 5.4. Managing patients' treatment expectations

According to Leventhal's Common Sense Model of Self-Regulation,^70^ patients develop cognitive lay representations of their illness and the treatment thereof. These representations guide coping strategies to manage health threats. Because of the heterogeneity in coping strategies (eg, avoidance, cognitive reappraisal, emotion venting, and seeking social support^50^) and their efficacy, individuals hold different expectations on being able to control their own health, which predicts stress.^79^ Therefore, it seems useful to offer patients the strategies to manage their expectations regarding treatment.^70^ This strategy was demonstrated in a case study^125^ whereby 2 patients were offered a side effect prevention training including psychoeducation about the disease and the treatment, adopted to patients' previous knowledge, promotion of doctor–patient communication, information about the nocebo effect, and conditioning processes involving the formation of associations between the medication and positive sensory experiences (eg, a beautiful song), and pleasant emotions and expectations by means of imagination exercises. Results indicated that a personalized approach improved treatment expectations and quality of life and reduced treatment side effects. For instance, greater self-efficacy was achieved by means of making coping strategies more concrete for the patient who had considerable previous knowledge and medical understanding. Conversely, the patients with less previous knowledge and who suffered from progressive anxiety benefitted more from psychoeducation aimed at the illness and its treatment. Such an individualized approach has been shown to be beneficial in previous research, in which individuals who regularly used avoidance as a coping strategy benefitted most from short and basic information, while anxious patients benefitted most from detailed information on positive treatment effects.^81,130^ Therefore, a possible avenue that aims at nocebo prevention could focus on optimizing coping strategies, based on the patient's needs, which can be identified by means of efficient, reliable, and valid screening instruments. These instruments may focus on risk factors such as fear of side effects and negative treatment expectancies.^81,125,130^

### 5.5. Selection of and tailoring treatment to patients at risk

Many pharmacological treatments come with considerable side effects, whether induced by the active medication or by negative treatment expectations. As these side effects have a negative impact on quality of life and could lead to treatment nonadherence, it is imperative to develop methods to prevent possible side effects from occurring.^5,28,65^ Therefore, strategies could be implemented that identify patients at risk and offer these patients enhanced side effect information in a manner that prevents the development of negative treatment expectations.^41,125^ Reliable and valid (web-based) screening instruments could be incorporated to identify patients at risk for nocebo side effects. These include personality traits,^45,106,122^ psychological distress,^5,76^ and negative previous experience with pharmacological treatments.^5,28,31,32^ Based on screening outcomes, (guided) tailored psychological trainings could be offered, which may include both generic components as well as treatment-specific side effect information that optimizes positive treatment expectations.^41^ An example of a possible intervention was developed for breast cancer patients undergoing endocrine therapy.^124^ The intervention included giving information on the treatment itself and the nocebo effect, guided imagination exercises that focused on control and positive expectations, optimizing coping skills by teaching problem-solving skills related to the most common side effects of endocrine therapy, reducing specific concerns by preparing cognitive and behavioral strategies, and optimizing coping skills regarding treatment expectations. Such tailored interventions may enhance positive treatment expectations, prevent or reduce fear of side effects, and provide effective coping strategies if (nocebo) side effects should occur.

### 5.6. Reversing the nocebo effect

Induction of nocebo effects can not always be prevented. Therefore, it is useful to be able to reverse nocebo effects once they are established. Several strategies exist that could be used to reverse acquired nocebo effects. One such strategy is extinction. In extinction, a conditioned stimulus that induces a nocebo effect is presented repeatedly without the negative associated stimulus (unconditioned stimulus), resulting in extinction of the nocebo effect over a number of trials.^4,44,57^ In a clinical setting, this would translate to presenting contextual cues of the treatment environment without any associated negative effects. However, it seems that, once established, nocebo effects are difficult to reverse.^19,20,32^ It is suggested that the mediating effect of anxiety and autonomic arousal may inhibit the learning of new associations.^19^

Another, less studied mechanism that may reverse nocebo effects is counterconditioning, in which a conditioned contextual cue is still present, but is now associated with a positive unconditioned stimulus.^62,96,117^ For instance, a study^7^ showed that nocebo effects of itch can be minimized and even reversed by means of counterconditioning in combination with verbal suggestions. In the first part of the study, negative expectations on itch were induced in healthy adults by means of verbal suggestions and a conditioning procedure. In the second part, positive expectations were induced again by means of verbal suggestions in combination with a conditioning procedure, or an extinction procedure was applied. Turning previously negative learned associations into positive associations not only significantly reduced nocebo itch but actually completely reversed the effects, indicating a placebo effect. Finally, a study by Benedetti et al.^15^ showed that verbal suggestions of analgesia and hyperalgesia on induced ischemic arm pain alone can completely counteract the effects of a conditioning procedure in healthy adults. The same study also showed that verbal suggestions on motor improvement or worsening also counteracted a conditioning procedure in patients with Parkinson disease. These studies indicate that, while extinction learning may not be sufficient to totally reverse acquired nocebo effects, counterconditioning and verbal suggestions may be powerful strategies to minimize and even reverse the nocebo effect in clinical populations.

## 6. Conclusion

This narrative review provides an overview of possible factors that contribute to the development of the nocebo effect in pain and related conditions and could therefore result in increased side effects, reduced treatment efficacy, and reduced quality of life. However, various opportunities exist that could prevent or minimize the occurrence of the nocebo effect, such as managing patient expectations by offering enhanced treatment and side effect information, optimizing communication style of health care providers and their relationship with patients, and providing psychoeducation on coping skills to manage patient expectations. Strategies may also be applied to extinguish nocebo effects once they are established, such as counterconditioning. Although these strategies may be used to alter the nocebo effect, more research is needed on its underlying mechanisms to identify possible factors that contribute to the susceptibility and develop more therapeutic approaches that can minimize nocebo effects in clinical settings. Furthermore, although there exists considerable knowledge of underlying mechanisms of nocebo learning, more research is needed on the application of this knowledge in various clinical (pain) populations.

## Disclosures

The authors have no conflict of interest to declare.

This study was funded by a European Research Council Consolidator Grant 2013 (ID: ERC-2013-CoG-617700_EXPECT HEAL-TH), granted to A.W.M. Evers. The funder had no role in study design, data collection and analysis, decision to publish, or preparation of the manuscript.
